# Evaluation of Functional and Radiological Outcomes of Long Bone Fractures in Non-union Treated With Nail and Plate With Osteo-Periosteal Flaps

**DOI:** 10.7759/cureus.73170

**Published:** 2024-11-06

**Authors:** Anteshwar Birajdar, Sushant Kumar, Rahul Salunkhe, Mukesh O Phalak, Tushar Chaudhari, Sagar Gurnani, Sarthak Walia, Archit Gupta

**Affiliations:** 1 Orthopaedics, Dr. D Y Patil Medical College, Hospital and Research Centre, Dr. D Y Patil Vidyapeeth (Deemed to be University), Pune, IND

**Keywords:** bone graft, bone nails, bone plates, fracture healing, fractures, non-union, osteo-periosteal flaps

## Abstract

Objective: To evaluate the functional and radiological outcomes of long bone fractures in non-union cases treated with nail and plate with osteo-periosteal flaps.

Methods: This prospective study included 20 patients with non-union long bone fractures treated at Dr. DY Patil Medical College, Pimpri, Pune over a two-year period. Patients underwent surgical intervention using nail and plate with osteo-periosteal flaps. Outcomes were assessed using the Non-Union Scoring System (NUSS), Radiographic Union Scale in Tibial Fracture (RUST), and Lower Extremity Functional Scale (LEFS) at regular intervals over one year. Time to union and complications were also recorded.

Results: The mean age of patients was 41.5 ± 7.1 years, with 60% being male. Femur fractures were most common (40%), followed by tibia (35%) and humerus (25%). The mean duration of non-union before treatment was 4.15 ± 1.7 months. The average time to union was 19.8 ± 3.9 weeks. NUSS scores decreased from 40.9 ± 8.06 at one week to 16.6 ± 8.2 at one year. RUST scores improved from 4.0 ± 0 to 11.3 ± 0.92, and LEFS scores increased from 9.6 ± 1.9 to 69.2 ± 5.1 over the same period. Complications occurred in 40% of cases, with surgical site infection being the most common (20%).

Conclusion: The use of nail and plate with osteo-periosteal flaps for non-union long bone fractures demonstrated improvements in both functional and radiological outcomes over a one-year follow-up period. However, careful monitoring for complications is necessary. Further studies with larger sample sizes are recommended to confirm these findings.

## Introduction

Long bone fractures, particularly those involving the femur, tibia, and humerus, are common injuries encountered in orthopedic practice. While most fractures heal uneventfully with appropriate treatment, a subset of these fractures may progress to non-union, a complication characterized by a failure of the fracture to unite and form a solid bony union within a reasonable time frame [1]. Non-union can lead to significant disability, long term pain, and hampered quality of life for patients [2].

The incidence of non-union in long bone fractures varies depending on several factors, including the pattern of the fracture, status of soft tissue injury, adequacy of fracture reduction and stabilization, and factors related to the patient demographics such as age, comorbidities, and smoking status. The reported incidence of non-union ranges from 5% to 10% for femoral fractures, 5% to 15% for tibial fractures, and 2% to 10% for humeral fractures [3,4].

The management of non-union poses a challenging situation for orthopedic surgeons, as it often requires revision surgery and specialized techniques to promote fracture healing. Traditional treatment options include the use of intramedullary nails or plates for fracture stabilization, combined with bone grafting or bone graft substitutes to enhance the biological environment for fracture healing [5].

One emerging modality for the management of non-union is the use of osteoperiosteal flaps, which involve the transfer of vascularized bone and periosteum from a distant site to the non-union site. This technique aims to provide both biological and mechanical support for fracture healing by introducing viable bone, periosteum (a source of osteogenic cells), and a vascular pedicle to promote revascularization and new bone formation [6,7].

While the use of osteoperiosteal flaps has shown promising results in several case series and small studies, the overall functional and radiological outcomes of this technique in the management of non-unions of long bones remain to be fully explored. Additionally, the optimal surgical approach, including the choice of fixation method (intramedullary nailing or plating), and the potential advantages of combining osteoperiosteal flaps with other adjunctive treatments, such as bone grafting or bone morphogenetic proteins (BMPs), require further investigation [8,9].

We aimed to evaluate the functional and radiological outcomes of long bone fractures in non-union treated with intramedullary nails or plates in combination with osteoperiosteal flaps. By assessing patient-reported functional outcomes, radiographic evidence of fracture union, and complication rates, this study will contribute to the growing body of knowledge regarding the efficacy and safety of this surgical technique in promoting fracture healing and improving patient outcomes.

## Materials and methods

The study was conducted in the Department of Orthopedics at Dr. DY Patil Medical College, Pimpri, Pune over a period of two years from August 2022 to August 2024 (approval I.E.S.C./361/2022). It aimed to evaluate the functional and radiological outcomes of long bone fractures in non-union cases treated with nail and plate with osteo-periosteal flaps. Twenty patients were included in this hospital-based prospective study.

The study's inclusion criteria aimed to select suitable candidates for evaluating nonunion treatments. Eligible participants were aged 18 to 60, ensuring sufficient bone healing potential while minimizing age-related risks. They needed to meet the specific diagnostic criteria for nonunion and provide informed consent after being briefed on the study's details. Additionally, a thorough medical evaluation was required to confirm pre-anaesthetic fitness for surgery.

The exclusion criteria aimed to eliminate individuals with confounding variables or increased surgical risks. Patients with pathological fractures, local soft tissue infections, or infected nonunions were excluded, as these conditions could affect healing and treatment outcomes. Individuals with severe systemic diseases-such as major cardiac, hepatic, pulmonary, or renal conditions-were also excluded to ensure the study population could safely undergo surgery and recover without significant comorbidities.

The researchers collected demographic data including age and gender of the patients. They also recorded relevant medical history, noting any comorbidities such as diabetes and hypertension. For each case, the location and type of fracture were documented, categorizing them into femur, tibia, or humerus fractures, and further classifying them as comminuted, oblique, segmental, spiral, or transverse fractures.

The surgeon began the procedure by making an incision along the previous scar and carefully dissecting to expose the non-union site, taking care to protect the neurovascular structures. The non-union was identified and debrided of fibrous tissue to reveal healthy bone ends. The osteoperiosteal flap donor was marked 4 cm proximal and distal to the fracture site. If necessary, the intramedullary canal was reamed to allow for the insertion of a new intramedullary nail, which was placed using a guidewire to span the fracture and provide axial stability. A long locking plate was selected and positioned to bridge the non-union site, with screws inserted proximally and distally for secure fixation. Compression across the fracture was applied where needed, and autologous bone graft or substitutes were used to promote healing at the non-union site. The decortication osteoperiosteal flap was used to cover the fracture site as this creates living bone chips attached to the periosteum which enhances the healing of fracture non union. Fluoroscopy was employed to confirm correct alignment and implant positioning. After ensuring the construct was stable, the surgeon closed the wound in layers, placed drains as required, and completed the procedure. Postoperative radiographs were used to assess the fixation.

The time interval between the initial fracture and the surgical intervention was recorded for each patient. Additionally, the duration of non-union prior to treatment was noted. The surgical procedure involved the use of nail and plate with osteo-periosteal flaps, though specific details of the technique were not provided in the results section.

To assess outcomes, the researchers employed several standardized scoring systems. The Non-Union Scoring System (NUSS) was used to evaluate the severity and progression of non-union [10]. The NUSS score is a clinical tool used to predict the likelihood of non-union in long bone fractures. It considers factors like smoking, infection, soft tissue injury, and fracture gap, helping guide the management of at-risk patients. Scores were recorded at multiple time points: one week, three weeks, one month, three months, six months, and one year post-surgery. For tibial fractures, the Radiographic Union Scale in Tibial Fracture (RUST) was utilized to assess bone healing, with scores recorded at the same intervals as the NUSS [11]. The RUST score is a radiological assessment tool used to evaluate the healing of tibial fractures by examining the presence and quality of callus formation at four cortical sites (anterior, posterior, medial, and lateral) on X-rays. Each site is scored from 1 (no callus) to 3 (complete bridging), with a total score ranging from 4 (no healing) to 12 (complete healing).

Functional outcomes were measured using the Lower Extremity Functional Scale (LEFS) for lower limb fractures [12]. Like the other scoring systems, LEFS scores were recorded at regular intervals throughout the follow-up period. The LEFS is a patient-reported outcome measure used to assess physical function in individuals with lower limb conditions, including fractures. It consists of 20 questions about daily activities, each rated from 0 (extreme difficulty) to 4 (no difficulty), providing a total score that reflects the patient's functional ability.

The time to union was carefully monitored and recorded for each patient. Complications were also documented, including surgical site infections, wound dehiscence, and persistent non-union.

Data analysis involved calculating means and standard deviations for continuous variables such as age, time from fracture to surgery, duration of non-union, and time of union. For categorical data like gender, fracture location, and complications, frequencies and percentages were computed. The progression of NUSS, RUST, and LEFS scores over time was analyzed to evaluate the effectiveness of the treatment.

**Figure 1:**
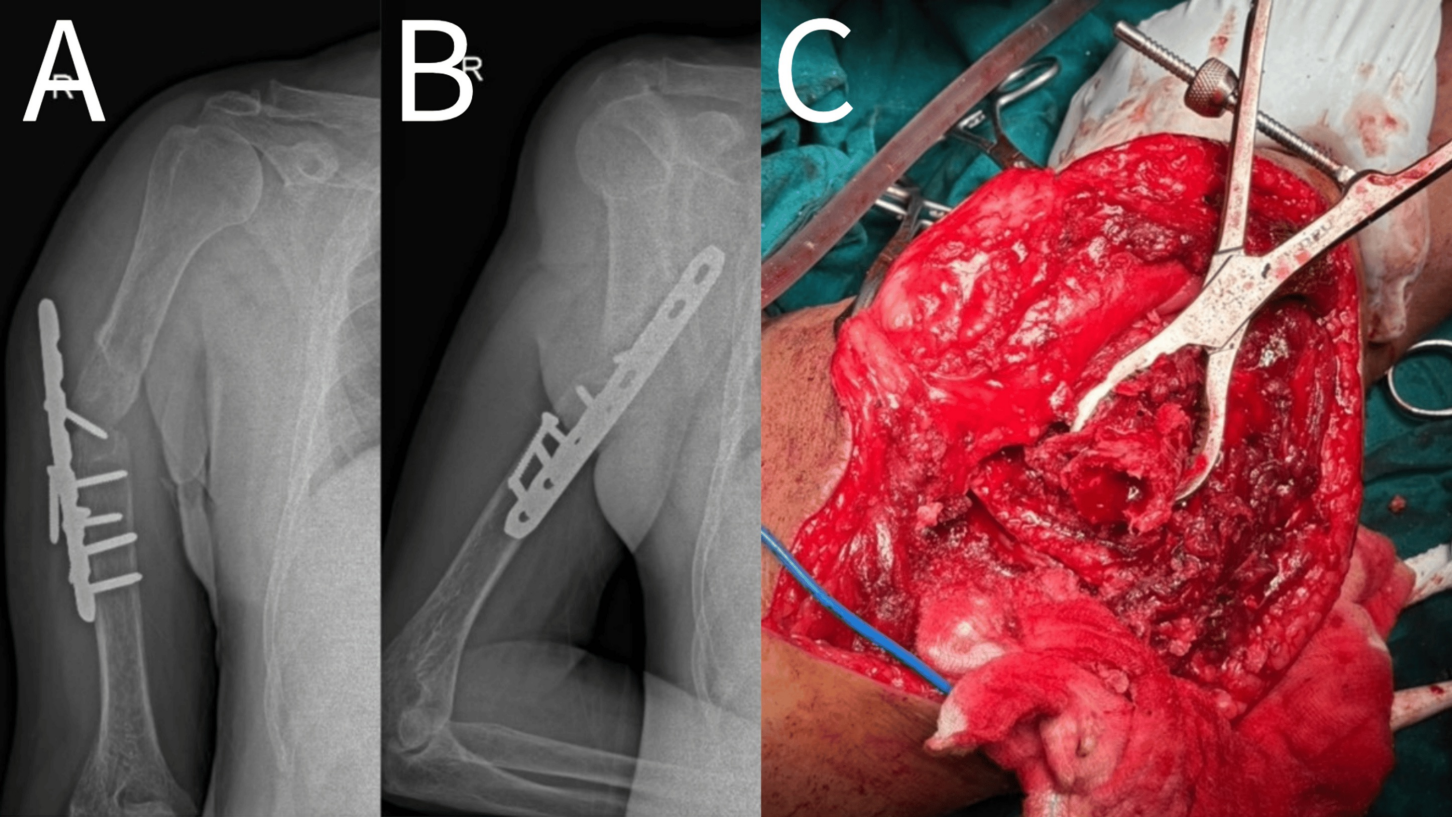
Pre-operative, intraoperative picture of a 53-year-old male showing humerus mid-shaft non-union with implant in-situ A - Anteroposterior view B - Lateral view C - Intraoperative image

**Figure 2:**
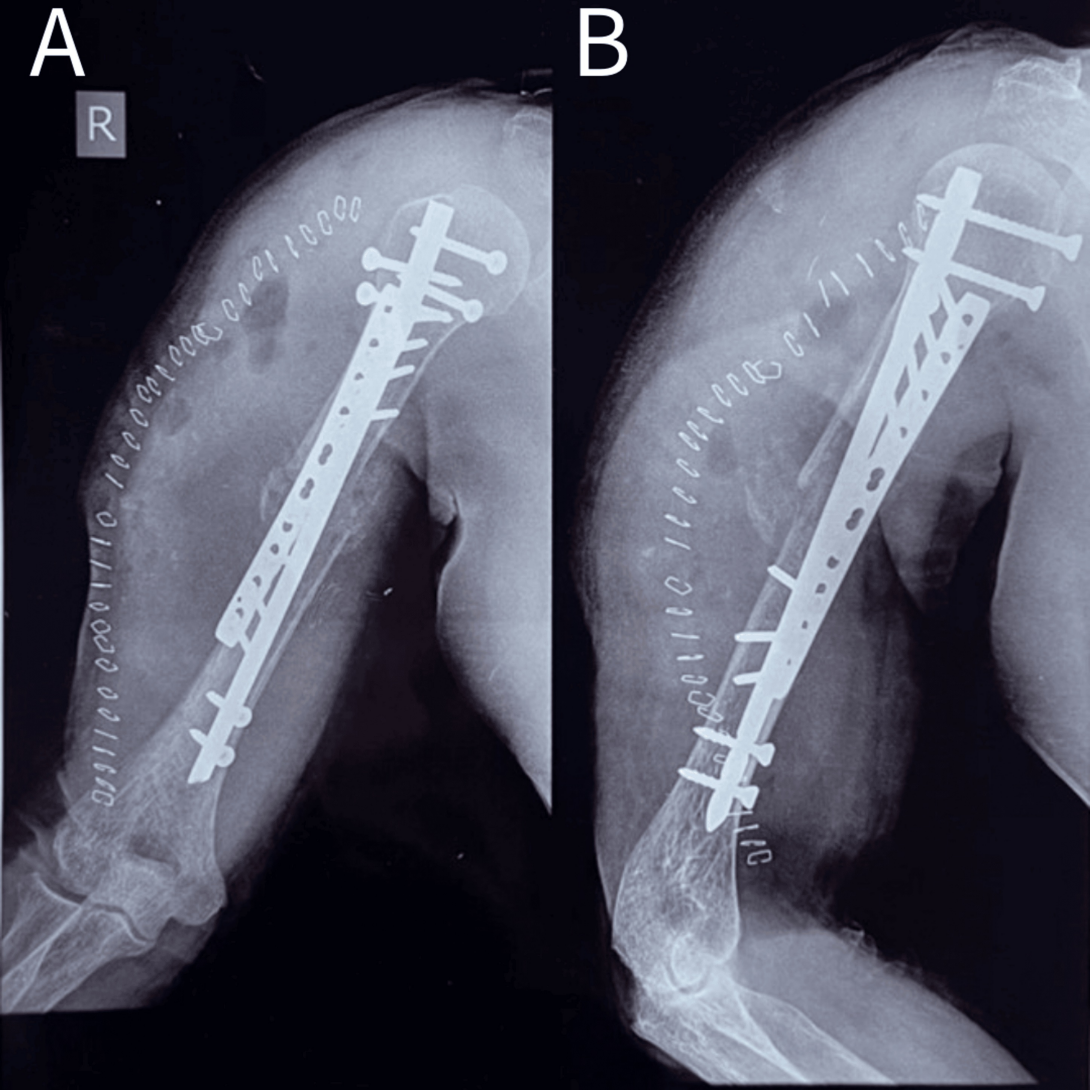
Post-operative picture showing a 53-year-old male with humerus union who underwent nail plate and osteoperiosteal flap A - Anteroposterior view B - Lateral view

The surgical procedure is shown in Figure 1 and Figure 2.

## Results

Table 1 shows the demographic characteristics of the 20 patients involved, with most patients being between 31-50 years old and a slight majority being male.

**Table 1:** Demographic Characteristics including age and gender

Characteristic		Frequency	Percentage
Age (in years)	20-30	2	10%
	31-40	7	35%
	41-50	7	35%
	>50	4	20%
Gender	Female	8	40%
	Male	12	60%

Table 2 outlines the clinical characteristics, revealing that femur fractures were most common, followed by tibia and humerus. Comminuted fractures were the most frequent type.

**Table 2:** Clinical Characteristics including co-morbidities, location of fracture and type of fracture

Characteristic		Frequency	Percentage
Co-morbidities	Diabetes Mellitus	2	10%
	Hypertension	3	15%
Location of fracture	Femur	8	40%
	Tibia	7	35%
	Humerus	5	25%
Type of fracture	Comminuted	7	35%
	Oblique	4	20%
	Segmental	2	10%
	Spiral	3	15%
	Transverse	4	20%

Table 3 provides details on surgical timing and non-union duration. On average, surgery occurred about 10 hours after fracture, with non-union lasting about four months before treatment. Union typically occurred around 20 weeks post-treatment.

**Table 3:** Surgical and Non-union Characteristics with mean time of union

Characteristic	Mean±SD	Range
Time from fracture to surgery (months)	9.9±2.6	6-15
Duration of non-union (months)	4.15±1.7	2-8
Time of union (weeks)	19.8±3.9	14-28

Table 4 displays the progression of three key outcome measures (NUSS, RUST, and LEFS scores) over time. Generally, NUSS scores decreased (indicating improvement), while RUST and LEFS scores increased (also indicating improvement) over the one-year follow-up period.

**Table 4:** Outcome Measures Over Time (Mean ± SD) NUSS - Non Union Scoring System (Range: 4-12) RUST - Radiographic Union Score for Tibial fractures (Range: 4-100) LEFS - Lower Extremity Functional Scale (Range: 0-80)

Time point	NUSS Score	RUST Score	LEFS Score
At 1 week	40.9±8.06	4±0	9.6±1.9
At 3 weeks	38.9±7.9	4.5±0.51	18.6±4.2
At 1 month	36.1±7.9	5.4±0.6	24.5±3.3
At 3 months	31.1±7.9	7.5±1.05	39.2±5.1
At 6 months	24.2±8.6	9.5±1.1	54.8±5.5
At 1 year	16.6±8.2	11.3±0.92	69.2±5.1

Table 5 shows that while 60% of patients had no complications, some experienced issues such as surgical site infection, wound dehiscence, or persistent non-union.

**Table 5:** Complications with their frequency

Complications	Frequency	Percentage
Surgical site infection	4	20%
Wound dehiscence	2	10%
Persistent non-union	2	10%
Absent	12	60%
Total	20	100%

## Discussion

Although prevalent, non-unions are challenging to deal with. The literature contains extensive data for the treatment of these non-unions. In the management of these non-unions, adjuvants and implant type are typically given precedence over soft tissue biology. The soft tissue dissection component is the main topic of this investigation. Inspite of its significance in contemporary hospital economics, not much is understood about the non-union population's epidemiology. It is acknowledged that there are notable differences in the non-union rates throughout fracture patterns, locations, and main treatment modalities.

The treatment of long bone non-unions remains a challenging problem in orthopedic surgery. Our study demonstrates that the combination of nail or plate fixation with osteoperiosteal flaps is an effective treatment strategy, resulting in high union rates and significant functional improvement.

Our study included 20 patients, with a majority (70%) between 31-50 years old. There was a slight male predominance (60%). Our study was similar to a study by Zura et al. on 767 non-unions found a mean age of 44.48 years, which aligns with your predominant age group of 31-50 years [2]. 

Femur (40%), tibia (35%), humerus (25%) were the long bones involved in our study. Santolini et al. reported that femoral and tibial non-unions are among the most common, which aligns with our findings [9]. Comminuted (35%), oblique (20%), transverse (20%), spiral (15%), segmental (10%) were the types of fracture in our study. 

Mean duration of non-union in our study is 4.15 ± 1.7 months. Fragomen et al. reported the time to union was 24.5 weeks (range 11-60) for non-unions treated with intramedullary nailing and bone grafting [13]. Testa et al. reported a mean time to union of 26 ± 13.7 weeks using the Ilizarov technique for tibial non-unions [14].

Our overall union rate of 90% at one year is comparable to or better than those reported in similar studies. When treated conservatively, fresh diaphyseal tibial fractures have demonstrated an excellent prognosis, with a non-union rate of about 1.1%. In contrast, nonunion rates in cases that are operated on are reported to be close to 5% in the literature [1,15]. In a similar vein, Judet and Patel's series of 1068 cases showed a 92% success rate. His study included 290 cases of aseptic nonunion with union rates of 94.8%, 126 cases of septic nonunion with union rates of 85%, and 108 cases of malunion with union rates of 98%. Within eight months, he reported a 99% union rate in his most recent sample of 297 instances [16]. In his case series, Ramoutar et al. demonstrated a 95% union rate when applying the Judet technique to treat nonunion of the upper and lower limb bones [17]. They also argued that when the Judet technique was applied correctly, the need for an autologous bone graft to treat the nonunion was reduced. In order to supplement the additional bone graft at the nonunion site, we took into consideration osteoperiosteal flap surgery. According to Kumar et al., union rates might range from 70% to 100%, contingent upon the patient's initial NUSS score at presentation [8]. In their case series of 20 patients with tibial fracture nonunion, Raju et al*.* reported a 100% union rate, but Guyver et al*.* found a 92% union rate in their study [18,19]. 

The classification of nonunions based on their severity or complexity has not been taken into account in either of these studies. According to a summary of the data by Tzioupis et al., nonunion rates range from 0% to 80%, depending on variables such co-morbidity, technique, and site [20].

LEFS scores improved from 9.6 ± 1.9 at one week to 69.2 ± 5.1 at one year. The improvement in LEFS scores from 9.6 to 69.2 over a year demonstrates significant functional recovery. When evaluating the lower limb, LEFS has demonstrated strong predictive correlation and reliability. Furthermore, in situations where there are tibia shaft fractures, it is a relevant and dependable method for tracking recovery [12,21]. Kumar et al. noted that the clinical improvement graph pattern as assessed by LEFS and the average time required for radiological union were comparable [8]. From the third month postoperatively, they found a strong correlation between the NUSS and RUST/LEFS scores. The association is most pronounced between the third and nine postoperative months. The lower the NUSS score, the better the union rate and the functional outcome as determined by the RUST and LEFS scores. Thus, the correlation analysis provides a good explanation for the prediction of the time to union based on the NUSS score.

Forty percent of patients experienced complications, including surgical site infection (20%), wound dehiscence (10%), and persistent non-union (10%). Guyver saw two patients with severe infections and three patients with superficial illnesses [18]. The infection rate in the Kumar et al. study was 20.5% (7/34), with 7% (1/14), 10% (1/10), and 50% (5/10) in group A, B, and C, respectively [8]. When comparing groups A and B, group C had a noticeably greater infection rate. This higher infection rate may have resulted from the factors we took into account when assigning an NUSS score. In every case, we were able to control the infection by using a prolonged course of antibiotics.

The use of osteoperiosteal flaps in our study may have contributed to the high union rates and favorable functional outcomes. This technique combines the benefits of mechanical stability provided by internal fixation with the biological enhancement of bone healing. Similar positive results have been reported by Weir et al. using vascularized periosteal flaps for recalcitrant non-unions [22]. Our technique used a combination of internal fixation using nails and plates which provides stability against the bending forces, axial loading and rotational forces that act at the fracture site. 

In a study by Kiran et al., the use of Ilizarov fixator was used to treat non union of humerus fracture, and pin site infection was seen in two cases (10.52%). They were superficial and were successfully treated by local cleansing and antibiotics. There was one case of radial nerve palsy seen in a patient where plate removal was done which recovered completely in three months time. Our method has the added advantage of lower chance of infection as compared to Ilizarov and other external fixation techniques [23].

Limitations and future directions

Our study is limited by its relatively small sample size and lack of a control group. A more thorough statistical analysis would have made the results more replicable. Future prospective, randomized studies comparing this technique with other established methods of treating non-unions would be valuable. Additionally, longer follow-up periods would help assess the long-term outcomes and potential late complications.

## Conclusions

In conclusion, our study suggests that the combination of nail or plate fixation with osteoperiosteal flaps is an effective treatment for long bone non-unions, with results comparable to or better than those reported in the literature. The technique appears to offer high union rates, significant functional improvement, and acceptable complication rates. Further research is warranted to definitively establish the optimal fixation method and to explore the full potential of osteoperiosteal flaps in enhancing bone healing in non-union cases.
